# Graphene-Based Materials in Dental Applications: Antibacterial, Biocompatible, and Bone Regenerative Properties

**DOI:** 10.1155/2023/8803283

**Published:** 2023-02-07

**Authors:** A. G. Williams, E. Moore, A. Thomas, J. A. Johnson

**Affiliations:** ^1^Department of Biomedical Engineering, University of Tennessee Space Institute, 411 B. H. Goethert Pkwy, Tullahoma, TN 37388, USA; ^2^Center for Laser Applications, University of Tennessee Space Institute, 411 B. H. Goethert Pkwy, Tullahoma, TN 37388, USA

## Abstract

Graphene-based materials have been shown to have advantageous properties in biomedical and dental applications due to their high mechanical, physiochemical, antibacterial, and stem cell differentiating properties. Although graphene-based materials have displayed appropriate biocompatible properties when used in implant materials for orthopedic applications, little research has been performed to specifically test the biocompatibility of graphene for dental applications. The oral environment, compared to the body, varies greatly and must be considered when evaluating biocompatibility requirements for dental applications. This review will discuss *in vitro* and *in vivo* studies that assess graphene's cytotoxicity, antibacterial properties, and cell differentiation ability to evaluate the overall biocompatibility of graphene-based materials for dental applications. Particle shape, size, and concentration were found to be major factors that affected overall biocompatibility of graphene.

## 1. Introduction

**Figure 1:**
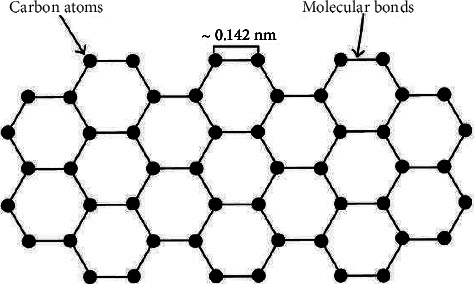
Depiction of a typical sheet of graphene (reprinted from Roberts et al. [1] following the Creative Commons Attribution (CC BY) license (https://creativecommons.org/licenses/by/4.0/) (accessed on 4 May 2022)).

Carbon is used in almost every field of science to some degree due to its abundance and variety of different structures it can form. Carbon is able to form many different structures due to its four valence electrons' ability to hybridize in different ways [1]. These electrons typically hybridize in one of three ways: sp, sp^2^, and sp^3^. One such structure that carbon can form is graphene, which is a two-dimensional structure of carbon atoms and one of the strongest materials in existence [2, 3]. Graphene was first isolated in 2004 by Novoselov et al. [4], and it consists of two-dimensional sheets that are made up of sp^2^ hybridized carbon atoms bonded together in hexagonal structures as seen in Figure 1. These sheets are typically less than 10 nm thick. Graphene has many properties that make it attractive for use in a wide variety of scientific fields such as high mechanical strength, electrical conductivity, and high surface area [5–7]. The two main derivatives of graphene are graphene oxide (GO) and reduced graphene oxide (rGO) [8–11].

Graphene and its derivatives have been studied for potential use in biomedical applications such as drug delivery, tissue engineering, and biosensors [12–14]. GO and rGO have been extensively studied for such applications due to their increased hydrophilicity and stability in the body when compared to graphene [10, 15]. One area where graphene may excel over GO and rGO is in the field of nanoparticles. Graphene nanoparticles are of interest to researchers for biomedical applications; in particular they have a lot of potential for use in dentistry where they may have applications as filler material in dental adhesives, teeth whitening compounds, and dental membranes [16–18]. GO can also be chemically functionalized with a wide range of different molecules in order to tailor it toward specific applications [13, 14], and functionalized graphene oxide (FGO) is commonly used in the field of medicine. GO is often functionalized with molecules that improve solubility and biocompatibility and introduce other favorable aspects such as enhanced biodegradability and antimicrobial properties. In the field of medicine alone, research has been done on the use of FGO in drug delivery systems, wound treatment, tissue engineering, bone scaffolding, and biosensors [19].

The main challenge that the widespread use of graphene nanoparticles in dentistry faces is the biocompatibility of graphene nanoparticles [20, 21]. While there are studies that look at the biocompatibility of graphene nanoparticles, many of them are inconclusive [22–24]. Furthermore, there is a lack of studies on the long-term health effects of these nanoparticles in the body. This study will provide an overview of the current status of the use of graphene nanoparticles in dentistry and what steps need to be taken in order to improve their use in the future.

Nanoparticles are of great interest to many scientific fields now, and much is still unknown about how certain materials behave on the nanoscale. This is especially true when it comes to biomedical applications. Nanoparticles are generally considered to be any particle under 100 nm in size, and they tend to have different properties when compared to bulk materials [25]. One reason for this is the immense increase in the ratio of surface area to volume. Many nanoparticles are so small that all of their atoms are on the surface. The high surface area to volume ratio of nanoparticles means that surface properties are far more important than bulk properties [26]. Many material properties change at the nanoscale such as mechanical strength, melting points, and optical properties [27]. There is still much that is unknown about the exact mechanisms behind some of these changes [28].

**Figure 2:**
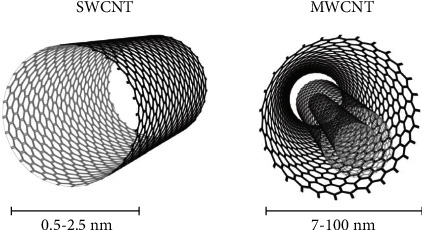
The shape and size of a single-walled carbon nanotube and a multi-walled carbon nanotube (reprinted from Ribeiro et al. [29] following the Creative Commons Attribution (CC BY) license (https://creativecommons.org/licenses/by/4.0/) (accessed on 4 May 2022)).

**Figure 3:**
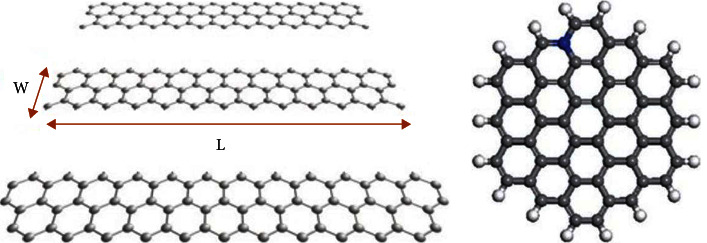
Typical shape of (a) graphene nanoribbons and (b) a graphene quantum dot [32, 33] (reprinted following the Creative Commons Attribution (CC BY) license (https://creativecommons.org/licenses/by/4.0/)).

Graphene nanoparticles can take a variety of shapes. The two most common shapes are flat sheets of graphene and graphene nanotubes. Flat sheets of graphene are simply small pieces of a single layer of a graphene sheet, while graphene nanotubes, also known simply as carbon nanotubes, consist of rolled up sheets of graphene as shown in Figure 2. A single-walled nanotube consists of a single rolled up sheet of graphene while multi-walled nanotubes can be made up of several layers of graphene [24, 30]. The simplest structures are shown in Figure 2, but the graphene sheets can be rolled in different directions to produce more complicated nanotube configurations. Additionally, graphene can exist in some more unique configurations such as nanoribbons, quantum dots, and other specific shapes such as pyramids [31]. Graphene nanoribbons and quantum dots are both graphene structures that are typically less than 100 nm in size (Figure 3), with nanoribbons being more elongated and quantum dots being rounder in shape [34]. Both configurations have enhanced electronic properties due to their smaller sizes which make them useful in fields such as quantum electronics [35].

Nanoparticles have some concerns when it comes to biocompatibility. Unlike bulk materials, nanoparticles are able to interact with and even enter individual cells. Additionally, nanoparticles are able to easily traverse the body through the bloodstream [36]. As noted earlier, nanoparticles have different properties when compared to the bulk form of the same material. This leads to biocompatibility testing being required for the nanoscale form of materials that have previously been tested in bulk form. Currently, there are still many unknowns surrounding the biocompatibility of nanoparticles in the body and what steps need to be taken to ensure they are safe to use.

Biocompatibility is one of the most important aspects of any material being used in a biological environment or application. The biocompatibility must be proven through extensive testing both *in vitro* and *in vivo* [30, 37]. Cell viability and proliferation assays are commonly used for *in vitro* biocompatibility assessments. Colorimetric MTT (3-(4,5-dimethylthiazol-2-yl)-2,5-diphenyltetrazolium bromide) assays are the most common assays to evaluate the cytotoxicity of dental materials since they are inexpensive and rapid [38]. Lactate dehydrogenase (LDH) assays are also used for cytotoxicity studies. LDH assays measure the cytosolic enzyme lactate dehydrogenase released by cells when significant membrane damage occurs, and the amount of LDH released is proportional to lysed cells [39]. Cell counting kit-8 (CCK-8) assays are used to determine cytotoxicity, cell viability, and proliferation of cells [40]. Hemocompatibility, genotoxicity, and sensitization tests can also be performed both *in vitro* and *in vivo* when assessing biocompatibility of dental and biomedical materials [38]. Murine models are among the most common forms of *in vivo* studies for biomedical research [41]. For dental materials, *in vivo* studies are often utilized for bone tissue engineering applications for regenerative medicine to treat craniomaxillofacial bone deformities, dental pulp regeneration, and periodontitis [27]. Rat calvaria defect models are often employed to determine osteogenic effects of materials, and subsequent micro-CT and histopathology tests are performed to determine bone regenerative properties and biocompatibility in comparison to surrounding tissues [42–44].

When testing the biocompatibility of a material, the location in the body itself must be taken into consideration. One issue when it comes to applications in dentistry is that the mouth has different biological considerations when compared to other parts of the body [45]. The pH level of the mouth is lower than that of the rest of the body, with studies showing an average pH of 6.7. Additionally, the pH of the mouth is highly variable. Different foods and beverages can alter the pH of the mouth during consumption. The pH level must be considered when performing biocompatibility tests as certain materials may erode at lower pH levels. Another important factor when discussing the mouth is the high pressure experienced by the teeth. The average force exerted by humans while chewing food is around 70 pounds per square inch. For people that grind their teeth in their sleep, that value can grow as high as 700 pounds per square inch [46]. Any material that is going to be integrated with teeth needs to be able to withstand such forces without breaking apart. With these considerations in mind, one major problem for dentistry is that many materials have been approved by the FDA for use in dentistry while only having biocompatibility testing done for other areas of the body.

As graphene-based materials are becoming popular in the field of dentistry, it is imperative that the biocompatibility of these materials is thoroughly tested. There has been extensive testing on the biocompatibility of graphene itself. The toxicity of graphene is mainly caused through physical contact between the graphene particles and the cell membranes as well as through the generation of reactive oxygen species (ROS) [37, 47, 48]. It is theorized that the initial toxicity levels depend mainly on the physical contact while the long-term toxicity is due more to the oxidation effects [49]. Prolonged exposure to graphene can also cause long lasting damage through genotoxicity, where the graphene particles damage the genetic information of healthy cells [50]. Such damage can lead to genetic instabilities and mutations. It has been found through studies that the cytotoxicity of graphene is closely linked to the shape, size, concentration, and chemical state of the graphene sheets [51–53]. An example of this is that carbon nanotubes, which consist of rolled up sheets of graphene, were found to be more toxic than flat sheets due to the narrow structure being able to easily pierce cell membranes [54]. This trend follows for other graphene shapes, with the narrower structures being more toxic [55]. With regard to size, it was found that the cytotoxicity tends to increase as the size of the graphene particles decreases [56]. The smaller particles are able to more easily pierce cell membranes and cause damage to the nucleus. Additionally, higher concentrations of graphene sheets have been linked to an increase in the generation of reactive oxygen species [53].

Another concern is the distribution and excretion of graphene nanoparticles in the body. Free graphene nanoparticles are easily able to pass through various biological barriers and spread throughout the body via the bloodstream [57, 58]. Looking at a variety of studies, it can be seen that the distribution profiles of graphene nanoparticles are very inconsistent. Factors such as the size, shape, and even functional groups can alter how graphene nanoparticles disperse through the body [59]. The excretion of graphene nanoparticles from the body is still an area of intense study as the excretion mechanisms vary greatly depending on factors such as the size and shape of the nanoparticles [60]. Many smaller nanoparticles are able to be easily excreted through the urine or feces, while larger sized nanoparticles tend to accumulate in the liver and spleen for up to several weeks before being excreted. Overall, it can be determined that there is still a great deal of uncertainty when it comes to the biocompatibility of graphene nanoparticles due to how dependent the results are on the size, shape, and concentration of the nanoparticles. The biocompatibility of graphene nanoparticles requires additional extensive testing both *in vitro* and *in vivo* before any clear conclusions can be drawn regarding their use in dentistry.

### 1.1. Selection Process

**Figure 4:**
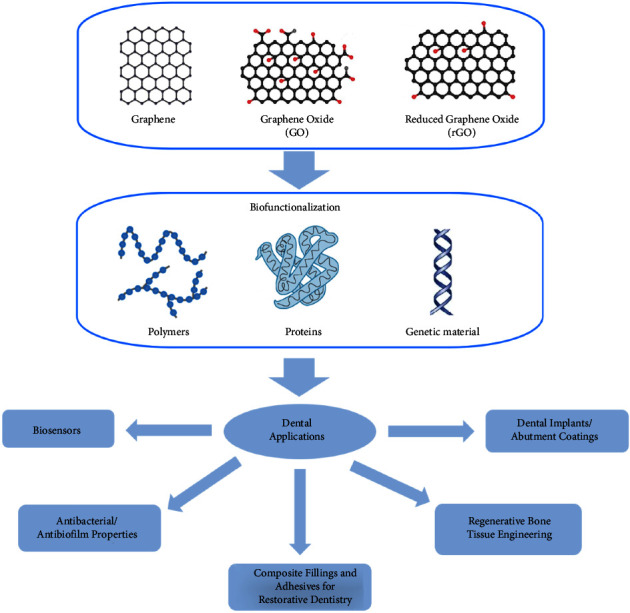
Graphical abstract of graphene-based materials for dental applications.

The process for choosing articles to review was very straightforward. Several databases were used for the search with the primary databases being Web of Science and PubMed. The keywords that were used in the search process were “graphene,” “nanoparticles,” “biocompatibility,” and “dentistry.” The majority of the articles selected come from the past five years in order to avoid overlap with previous review articles. Among the articles found, 69 research articles were reviewed that assessed graphene-based materials in various dental applications (Figure 4). The articles chosen incorporated at least one biocompatibility study including in *vitro/in vivo* assessment, cytotoxicity tests, MTT assays, and proliferation and differentiation tests that could be applicable to dental applications.

## 2. Antibacterial/Antibiofilm Properties of Graphene and Its Derivatives for Further Biocompatibility Enhancement in Dental Applications

One of the most common causes of dental implant failure in patients is infection of the gums with the most common being peri-implantitis and periodontitis. In fact, it is estimated that approximately 47.2% of adults aged 30 and older have some form of periodontal disease and approximately 28% of dental implant patients will acquire peri-implantitis infections [61]. While these infections can be managed, their main form of treatments include laser surgeries, tissue grafts, and more implants, as common antibiotics have proved to be ineffective. Although implant infections are a major problem, common issues like cavities are also caused by high amounts of bacteria. It is estimated that approximately 3.4 billion people worldwide are plagued with common bacterial infections such as periodontitis and dental caries (cavities) [62]. For these reasons, scientists have looked for other treatments to mitigate bacterial infections such as the use of antibacterial materials for implants [11, 63]. Many antibacterial materials exist such as metal ions/oxides, antibiotics, quaternary ammonium compounds, and antimicrobial peptides; however, many of these materials are either highly toxic, develop bacterial resistance quickly, or have very high costs. However, graphene has surfaced as a viable antibacterial material for its low costs, renewable capabilities, and its ideal optical, electrical, and physical properties [5, 64–67].

**Figure 5:**
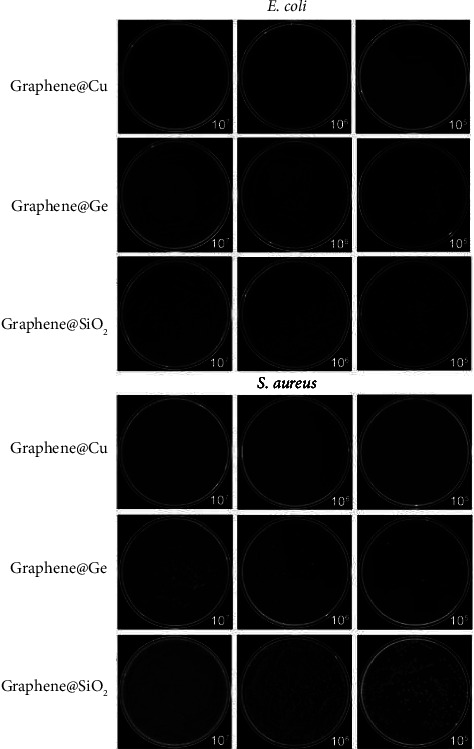
Photographs of *E*. *coli* (top) and *S*. *aureus* (bottom) cultured on the three types of graphene films (reprinted from Li et al. [77] following the Creative Commons Attribution (CC BY) license (https://creativecommons.org/licenses/by/4.0/) (accessed on 4 May 2022)).

Scientists have found many different mechanisms for graphene to achieve antibacterial activity. Firstly, graphene can physically damage the membrane of the bacteria by coming into direct contact with bacteria using its sharp edge [68]. This severely disrupts the cell membrane causing cell death. Secondly, graphene can chemically damage the cell by causing oxidative stress through the production of reactive oxygen species (ROS). The ROS then deactivates the proteins and lipids in the bacteria ceasing their ability to proliferate [69, 70]. Thirdly, graphene can disrupt the membrane of the bacteria through electron transfer. The graphene can act as an electron acceptor by removing electrons from the bacterial membrane and disrupting the membrane integrity resulting in cell death [71]. Other mechanisms exist as well, including interruption of the bacterial glycolysis process [72], damaging the DNA [73], growth prevention via graphene oxide sheet wrapping [15], and producing more ROS through the use of oxygen nanobubbles [74]. To investigate graphene's physical damage mechanisms, Pham et al. studied the use of pristine graphene nanosheets against bacterial infections and found that the nanosheets were able to exhibit bactericidal tendencies toward *P. aeruginosa* and *S. aureus* bacteria. The authors found that the density of the sharp edges of the bacteria was directly correlated to their antibacterial abilities. Other ways that graphene has been found to attack bacteria include oxidative stress, which inhibits the bacteria's ability to proliferate due to ROS production and electron transfer, which disrupts the bacteria's membrane by acting as an electron acceptor and taking electrons from the bacteria's membrane [11, 63, 75, 76]. Li et al. investigated the antibacterial activity of monolayer graphene films on Cu, Ge, and SiO_2_ where they found that the films were able to prevent the proliferation of *S. aureus* bacteria and *E. coli* on both the Cu and Ge materials but not SiO_2_ as can be seen in Figure 5. They believed it to be due to oxidative stress; however, there has not been an accurate technique to distinguish the exact cause of the antibacterial activity yet. However, they did conclude that the monolayer graphene films did possess moderate antibacterial activity [77].

**Figure 6:**
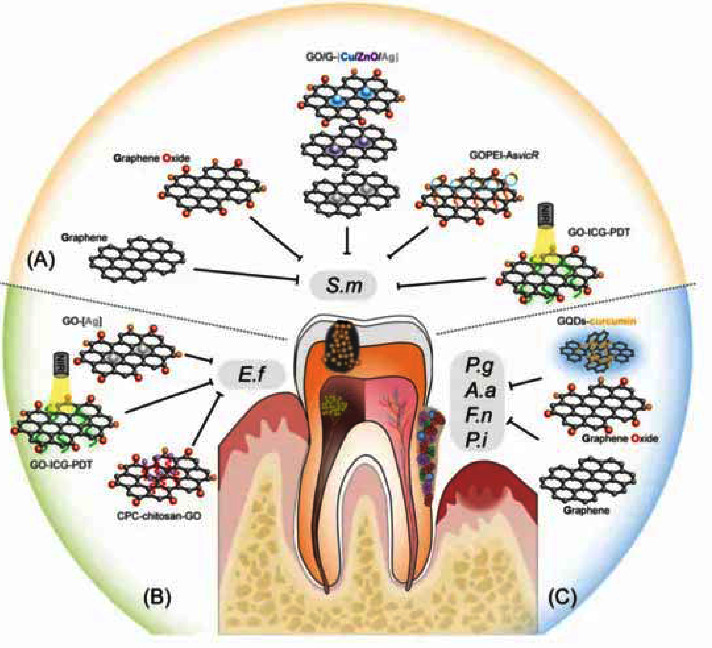
Graphical representation of different graphene materials that can inhibit oral bacteria (reprinted with permission from [78]) (Copyright 2015, American Chemical Society).

Additionally, graphene's derivatives and its combinations with other metals have been found to exhibit these antibacterial properties as seen in Figure 6. In fact, it has been found that graphene oxide has shown an exceptional ability in preventing caries, cariogenic bacteria, and tooth demineralization [78]. He et al. investigated graphene oxides' antibacterial activity against *S. mutans*, *P. gingivalis*, and *F. nucleatum,* which are common bacteria to periodontitis and caries, by using an MTT reduced assay, a CFU counting method, growth curve observations, and live/dead fluorescent staining. They also used TEM to investigate the physical structure of the cells and found that the nanosheets were able to disrupt the cell wall and membrane of the bacteria. Thus, they concluded that the graphene oxide nanosheets did serve as an effective antibacterial material against certain oral bacteria [78]. Other studies have been conducted evaluating the use of graphene and graphene oxide-based metal nanomaterials in preventing cariogenic pathogens and periodontitis bacteria. It has been found, in fact, that graphene, when coupled with certain metals, can increase their antibacterial properties along with their optical, electrical, and physical properties as well. Chen et al. studied how incorporating reduced graphene-silver nanoparticle nanocomposites into glass ionomer cements would affect their mechanical and antibacterial properties. They utilized direct contact tests (DCTs), scanning electron microscopy (SEM) observations, XTT assays, and bacteria live/dead assays to observe how the nanocomposites affected the structure and composition of *S. mutans* bacteria. They observed fewer amounts of live and dead bacteria in the cements with the nanocomposites and concluded that the nanocomposites did in fact improve the antibacterial activity of the dental cements [79].

As mentioned previously, dental caries is a very common bacterial dental condition that causes detrimental effects to the teeth. While the main cause of caries is in fact bacterial infection, dental caries is a predominantly biofilm-dependent disease which means anti-biofilm activity is just as important as antibacterial activity. Biofilms are large layers of microorganisms and bacteria that have attached together to form a specific extracellular matrix. This matrix is what makes it difficult for materials to disrupt these bacteria and thus treat conditions like dental caries. However, graphene and graphene oxide-based materials have been shown to exhibit both antibacterial and antibiofilm activity. Mao et al. explored how graphene-oxide copper nanocomposites affected the biofilm formation of *S. mutans* bacteria. Growth curves of different *S. mutans* bacteria were created, and the colony-forming unit counting method was used to identify the amount of viable bacterial cells. Cell cultures and several cell viability assays were used as well. The authors were able to find that the graphene-oxide nanocomposites did disrupt biofilm formation in *S. mutans* bacteria and had antibacterial effects with minimal cytotoxicity [80]. Kulshrestha et al. also investigated anti-biofilm properties of graphene by investigating how graphene/zinc oxide nanocomposites could affect cariogenic *Streptococcus mutans* and how coating this nanocomposite on artificial acrylic teeth could prevent biofilm formation. Through the use of cellular viability assays, biofilm formation assays, cytotoxicity assays, and several imaging techniques, the authors found that the graphene nanocomposite resulted in a significant reduction in biofilm formation and in the amount of the *S. mutans* bacteria. They also found a decrease in the overall toxicity of the artificial teeth with the addition of the graphene coating and accredited it to the reduction in biofilm formation [81].

**Figure 7:**
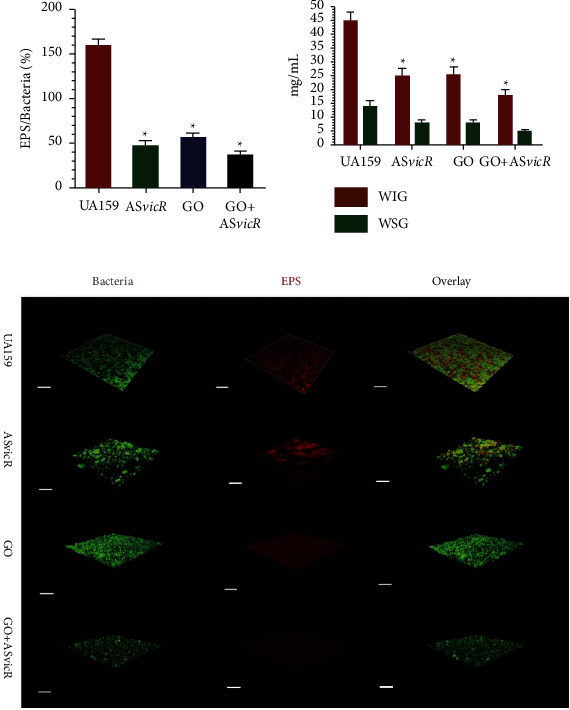
(a) Bar graph depicting the volume ratio of the exopolysaccharide (EPS) matrix to the bacterial biomass in the biofilms in different graphene complexes. (b) Bar graph depicting the water-insoluble glucan and water-soluble glucan levels of the different graphene complexes from different bacteria strains. (c) 3D reconstruction of the biofilms in different graphene complexes where green displays bacteria and red displays the EPS matrix (reprinted with permission from [83]) (Copyright 2020, American Chemical Society).

Not only can graphene be combined with metals and metal oxides, but they can also be used in combination with polymers. Graphene is commonly functionalized with polymers such as PLLA, PVC, CS, and PL to improve graphene's solubility and thus overall biocompatibility. Mazaheri et al. studied how incorporating graphene oxide into chitosan matrices will affect their antibacterial properties and ability to promote stem cell proliferation. Antibacterial tests were performed on *Staphylococcus aureus* bacteria along with cell viability assays and imaging techniques like AFM and SEM to test stem cell proliferation. From the results, the authors were able to find that the addition of the graphene oxide to the chitosan sheets resulted in a significant reduction in bacterial growth while still promoting the proliferation of mesenchymal stem cells. They concluded that the graphene oxide chitosan sheets could be used for antibacterial applications and could be used to promote tissue regeneration [82]. This is quite promising as one of the major issues in the oral bacterial infections in gum tissue degradation. Graphene oxide polymer complexes have also been used to extend graphene's antibacterial capabilities to viral infections. Wu et al. created a graphene oxide plasmid transformation system using interacting graphene oxide-polyethylenimine (PEI) complexes that are loaded with an AsvicR-expressing plasmid (GOPEI-AsvicR) in order to investigate how it will affect the viability of *S. mutans* bacteria and the production of virulence-associated genes. After several characterizations, the authors found that the graphene oxide complex did slow down the proliferation of the *S. mutans* bacteria, and that the AsvicR expression resulted in a reduction of the virulence-associated genes as seen in Figure 7. They concluded that this complex could not only have antibacterial and antiviral properties but also antibiofilm applications [83]. It has also been found that functionalizing graphene oxide sheets with polyvinylpyrrolidone (PVP) that has been conjugated with silver nanoparticles can improve graphene's antimicrobial and biocompatible properties. The silver nanoparticles themselves possess antibacterial properties as well by producing ROS that kill bacteria and exhibit lower resistance development. From the addition of silver nanoparticles on the graphene-PVP functionalized sheets, the entire composite possesses a higher affinity for killing bacteria and overall increases biocompatibility. Khalil et al. investigated these graphene oxide sheets with PVP conjugated with silver nanoparticles and cultured them with normal skin fibroblasts and several different bacteria to investigate their antimicrobial and biocompatibility properties. They found that the addition of the silver nanoparticles to the PVP graphene oxide sheets improved cell viability by approximately 40% and exhibited effective microbial growth inhibition killing about 60% of bacteria. This study proves that the addition of silver nanoparticles to polymer graphene oxide complexes can significantly improve graphene's antimicrobial and biocompatible properties [84].

As mentioned previously, bacterial infections are considered an ongoing, immense concern for our society as many bacteria have become immune to the everyday medications prescribed by doctors. To mitigate this, other avenues have been investigated for treating these bacterial infections such as graphene complexes that utilize specific features such as sharp edges to puncture the bacteria or chemical releases of ROS to kill the bacteria. However, if bacterial infections are identified too late, then these treatments are rendered almost useless. Therefore, the early detection of these bacterial species in or around the body is just as important. Therefore, further research has been conducted on graphene for use as a bacterial biosensor. These biosensors can be placed onto the enamel of teeth or various dental tools to detect the presence of harmful bacteria and thus prevent the start of an infection. Graphene presents as an ideal candidate for this due to its many properties such as high electronic and thermal conductivity, intrinsically high surface to volume ratio, and its chemical inertness. A large surface area allows for optimal interaction with biomolecules allowing for a high number of molecules to be detected. The basis of the sensors works on the principle that there is a change in the conductivity of the graphene when different bacterial species adhere to its surface. It has been found that different bacteria along with differing growth/adhesion patterns can yield various conductivity changes allowing for specific bacteria detection. Mannoor et al. investigated the creation of a wireless graphene nanosensor functionalized with antimicrobial peptides via silk bioresorption. The graphene-based sensing element with a wireless readout coil was generated on silk fibroin, and then the ultra-thin nanosensors were bio-transferred from the silk platform onto tooth enamel, via dissolution of the supporting silk film. The high surface area of the graphene allowed for high conformability to the biomaterial surface and improved contact with bacteria. They found that the sensors had high sensitivity, good biorecognition, and good conformability to the tooth enamel surface due to the graphene and incorporated peptides. Therefore, they concluded that they had successfully created a graphene nanosensor that could detect bacteria down to a single bacterium on tooth enamel while wirelessly reading out the results [85]. This study shows the numerous properties of graphene that allow it to successfully identify bacteria while achieving good contact with biomaterials without the hindrance of its electrochemical properties and any cytotoxic effects. Other research has been conducted to further investigate the capabilities of graphene for use in biosensors such as nonfunctionalized graphene biosensors, 3D microstructure biosensors, and even microfluidic chemiresistive biosensors. All have found that the graphene has allowed for precise bacterial detection on or in mediums such as saliva, tooth enamel, various biological tissues, and biomedical equipment [86–90].

## 3. Graphene in Composite Fillings and Adhesives for Restorative Dentistry Applications

Various dental materials such as resin composites and cements are used as filling materials for restorative dentistry in areas of infected teeth or cavities. However, if the material is not biocompatible and properly tested before use, it can harm the tooth and surrounding soft tissue, leading to hypersensitivity and further tooth decay [37]. This section will discuss the various uses of graphene in dental composites for tooth cements, filler materials, and adhesives and summarize the biocompatibility of graphene in such applications.

**Figure 8:**
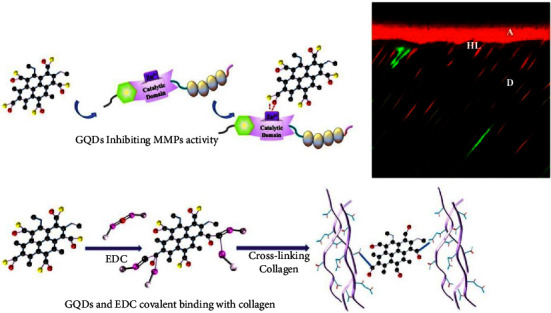
Schematic of graphene quantum dots and EDC inhibiting MMP activity to decrease dentin demineralization (reprinted with permission from Chen et al. [91]) (Copyright 2021, Elsevier).

Dental composites are commonly used as restorative filling material to replace decayed or infected areas of the tooth. Dentin demineralization occurs at the infected site, so composite cements are needed to restore the dentin and prevent further decay from present bacteria that may persist in the area. In addition, a main cause for resin-dentin bond failure is from the hybrid layers degrading collagen fibrils by activating host-derived matrix metalloproteinases (MMPs) in the dentin matrix. In order to inhibit degradation of collagen fibrils, researchers fabricated graphene quantum dots with 1-ethyl-3-(3-dimethylaminopropyl) carbodiimide (EDC) to modify the resin-dentin bone interface and increase the durability of such dental bonding material. The graphene quantum dots inhibited collagenase activity and MMPs by covalently linking collagen fibers to lessen enzymatic hydrolysis of collagen fibers (Figure 8) [91]. From the results, the researchers found promising results and good biocompatibility. Another group created a composite fabricated with nanohydroxyapatite and multi-walled carbon nanotubes and graphene oxide (nHAP/MWCNT-GO) to cover and protect dentin from demineralization. The coating assisted in decreasing erosion of dentin by forming an acid-resistant surface film [92]. GO has also been functionalized with various nanoparticles such as silver, calcium fluoride, and tricalcium phosphate to prevent dentin decalcification. The researchers found that when GO was conjugated with silver and silver-calcium fluoride, inhibition of *S. mutans* bacteria was displayed, and the composite exhibited low cytotoxicity except at higher concentrations of about 0.1 w/v% [93].

Graphene has been used in various types of dental resins to strengthen bonding and adhesive strength in restorative dentistry. Dental resins assist in bonding dental composite materials to hard tissues of teeth to treat dental caries or cavities that are caused by tooth decay. However, if the site it not properly sealed, bacteria can easily access the cured dental tissues through cavities at the tooth restoration interface. In addition, adhering materials onto dentin is challenging since dentin has higher water content compared to enamel and is less mineralized [94]. Therefore, research into alternative materials to act as fillers for dental adhesives is required. Graphene nanoplatelets have been studied for antimicrobial and antibiofilm properties and combined with polymer materials to act as better dental adhesive [95]. From the study, the graphene nanoplates inhibited the growth of *S. mutans* bacteria *in vitro* and demonstrated good mechanical performance without decreasing adhesive strength. Reduced nano-graphene oxide and graphene nanoplates doped with silver nanoparticles were prepared by another research group, which found that the graphene nanoplates doped with silver nanoparticles displayed good adhesive properties that improves the resin-bonded dentin interface. In addition, cell viability of the adhesive was found to be above 85% [96]. Another study found that GO paired with hydroxyapatite strengthened the durability and remineralization of resin-dentin bonds as well as improved adhesive properties [97].

Dental resins and cements are commonly employed in orthodontic applications. Orthodontic bonding resins have been made by mixing fluorinated graphite and bioactive glass to improve antibacterial properties and increase remineralization effects to aid in preventing white spot lesions on the enamel surfaces [98]. Silanized GO (SGO) nanoparticles have also been used in orthodontic bracket adhesives and dental adhesives, and researchers found that the addition of 0.25 wt% of SGO in commercial Transbond XT adhesive displayed excellent antimicrobial and mechanical properties. No significant difference in cell toxicity of human gingival fibroblasts was found between the control Transbond XT adhesive and the SGO modified adhesive, thus indicating that the addition of SGO nanoparticles did not affect overall cytotoxicity of the material [99].

Functionalized graphene and hydroxyapatite fillers were used as reinforcing particles for light-cured adhesives for dental applications. Silver-doped hydroxyapatite (HA-Ag), silver-doped graphene (Gr-Ag), and graphene and silver-doped hydroxyapatite (HA-Ag-Gr) nanofillers were investigated as main components in bis-GMA (2,2-bis[4-(2-hydroxy-3-methacryloxypropoxy) matrices for resin-based dental adhesives. The authors found that the experimental samples had good bond strength when compared to the control at earlier stages of aging when compared to the control Clearfil SE BOND 2 adhesive. Cytotoxicity studies were performed with a WST-1 (4-[3-(4-iodophenyl)-2-(4-nitro-phenyl)-2H-5-tetrazolio]-1,3-benzene sulfonate) colorimetric cell proliferation assay using human gingival fibroblasts (HGF-1) to test local biocompatibility up to 6 months *in vitro*. From the results, the authors found that the experimental adhesives showed no significant cytotoxicity over a 6-month time period based on ISO 10993-5 cytotoxicity standards that state that a medical material has cytotoxic potential if the cell viability is lower than 70% compared to negative controls used in the experiment [100].

**Figure 9:**
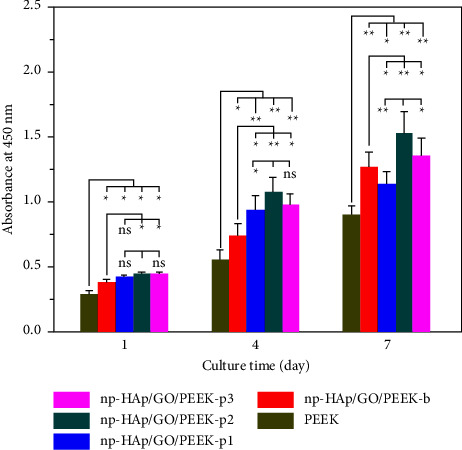
Cell proliferation results for MC3T3-E1 cells cultured onto the surface of PEEK nanofillers: np-HAp/GO/PEEK-b, np-HAp/GO/PEEK-p1, np-HAp/GO/PEEK-p2, and np-HAp/GO/PEEK-p3 composites (^*∗*^*p* < 0.05; ^*∗∗*^*p* < 0.01; *n* = 9 in each group) (reprinted with permission from [101]) (Copyright 2021, Elsevier).

Another study was conducted to improve polyetheretherketone (PEEK) nanofillers for bone restoration in orthopedic and orthodontic/dental applications. The researchers created novel surface-porous nanofillers made of PEEK combined with hydroxyapatite (HA) and graphene oxide via a heated injection mold process. The surface morphology, mechanical properties, and cellular responses of the nanofillers were investigated. It was found that the inclusion of the hydroxyapatite and graphene oxide significantly improved the overall cell adhesion and proliferation on the PEEK surface showing notable promise in improving tissue integration of PEEK nanofillers for orthopedic and dental/orthodontic applications (Figure 9). The authors performed in vitro cell studies with MC3T3-E1 cells to evaluate the effects of GO and HA inclusion and porous surface structure on cell adhesion and proliferation. From the studies, the authors found that the composites have overall good cell proliferation, and from CCK-8 results, composites with added HA and GO displayed significantly higher cell viability when compared to bare PEEK material. This further shows that the many qualities of graphene oxide, especially when combined with hydroxyapatite, are ideal for improving the biocompatibility and functionality of dental resins and composites [101].

From these studies, graphene oxide and graphene-based materials are promising as additional filler materials for uses in dental composites and resins in restoratives dentistry and orthodontics. The addition of graphene-based materials as fillers has been shown to provide good mechanical performance in dental composites. Graphene-based materials have also shown enhanced bonding strength in dental adhesives, offer good protection from dentin demineralization in infected teeth, and possess good biocompatibility in such dental applications.

## 4. Graphene-Based Materials in Regenerative Bone Tissue Engineering

**Figure 10:**
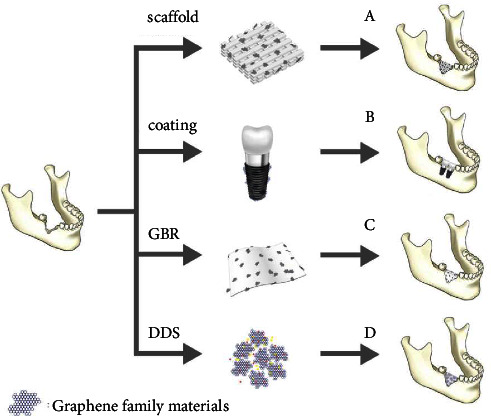
Graphene-based materials used in (A) scaffold materials for bone regeneration, (B) coatings on dental implants to promote osseointegration, (C) bone membrane regeneration acting as a guide, and (D) drug delivery systems (DDSs) to induce bone regenerative properties (reprinted from Cheng et al. [112] following the Creative Commons Attribution (CC BY) license (https://creativecommons.org/licenses/by/4.0/) (accessed on 4 May 2022)).

Osseointegration and differentiation of stem cells are important factors to consider when creating biomaterials for restorative dental applications and bone tissue engineering applications. Graphene has previously been used in various bone tissue engineering applications to promote cell attachment, proliferation, differentiation, and osteogenesis for dental applications. Graphene-based materials also possess optimal tissue engineering properties by having high surface area, mechanical strength, and ease of functionalization [102, 103]. Various methods to create graphene-based materials for bone tissue engineering have been explored such as electrospinning [64, 104, 105], freeze drying methods [106], chemical vapor deposition [107–109], 3D printing [110], and solvent casting [111]. This section will explore studies that included graphene materials in dental bone tissue engineering to promote differentiation of related stem cells and promote better osseointegration (Figure 10). In order to be considered for *in vivo* applications, biocompatibility of materials must be assessed. Therefore, the following studies included *in vitro* biocompatibility tests to further assess graphene's biocompatibility for potential dental applications.

Understanding and controlling mesenchymal stem cell (MSC) differentiation mechanisms is an important area of research for tissue engineering. One study investigated the effects of graphene and graphene oxide substrates on osteogenic differentiation of MSCs isolated from adult bone marrow. The chemical interaction between the substrates was investigated to determine molecular insights into proliferation enhancement mechanisms. From the results, higher osteogenic differentiation of MSCs was observed on graphene films in comparison to graphene oxide films. The authors found that the graphene films could preconcentrate osteogenic inducers, dexamethasone and Β-glycerolphosphate, which accelerate differentiation of MSC toward osteogenic differentiation [113]. Graphene nanogrids have also been created to accelerate differentiation of human MSCs isolated from umbilical cord blood (UCB) of an infant into osteogenic lineages. The nanogrids were fabricated from graphene nanoribbons that were synthesized from the unzipping of carbon nanotubes, and the nanoribbons were deposited on a PDMS substrate to form the nanogrid [73]. From the study, patterned proliferation of cytoskeleton fibers was comparable to unpatterned proliferation on hydrophilic GO sheets. Reduced graphene oxide nanogrids displayed the fastest osteogenic differentiation of human MSCs, and the amount of differentiation was around 2 times greater when compared to reduced graphene oxide sheets. The increased osteogenic differentiation of the reduced graphene oxide nanogrids could be attributed to the nanogrids' high adsorption of chemical inducers like ascorbic acid and the physical stress induced by the surface topographic features of the nanogrids.

Human MSCs isolated from an infant USB were used to evaluate the concentration and size-dependent cytotoxic and genotoxic effects of graphene sheets and nanoplatelets [53]. Reduced graphene oxide nanoplatelets (rGONPs) were synthesized and compared with rGO sheets to determine the size-dependent threshold concentration for cytotoxic and genotoxic effects of the graphene-based materials. From the study, rGONPs with average lateral dimensions of 11 ± 4 nm at 1 *μ*g/ml concentrations and rGO sheets with dimensions of 3.8 ± 0.4 *μ*m at high concentrations of 100 *μ*g/ml displayed high cytotoxic effects after 1 hr of exposure. The likely causes were due to the sharp edges from the graphene and oxidative stress created when interacting with cells. In addition, rGONPs could penetrate into the nucleus of human MSCs and cause genotoxicity at low concentrations around 0.1 to 1 *μ*g/ml after 1 hr.

**Figure 11:**
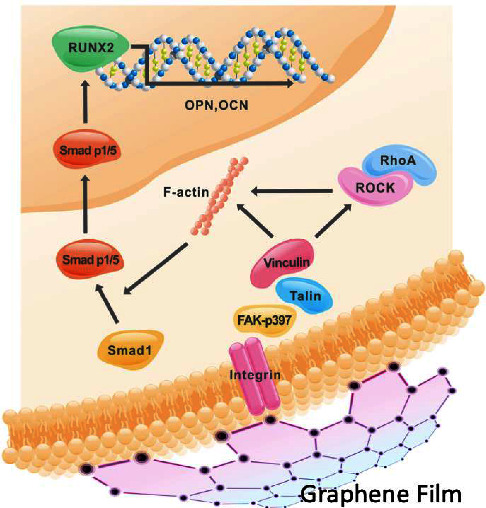
Integrin/FAK mechano-transduction pathway induced by graphene films (reprinted with permission from Xie et al. [109] following the Creative Commons Attribution (CC BY) license (https://creativecommons.org/licenses/by/4.0/) (accessed on 4 May 2022)).

Chemical vapor deposition (CVD) has shown promise in creating graphene films and scaffolds for tissue engineering. Dental pulp stem cells (DPSCs) are commonly used to test the biocompatibility, cell differentiation, and proliferation of graphene incorporated materials using a CVD method for dental applications. DPSCs possess a multi-lineage differentiation potential which allows them to differentiate into odontoblasts that assist in tooth dentin regeneration or osteoblasts which form bone matrices in the tooth [114]. In addition, GO-based substrates have been shown to increase DPSC attachment and proliferation and also increase the expression for RUNX2 proteins that are essential for osteoblastic differentiation [115]. One study used CVD to transfer graphene onto austenitic stainless steel (ASS). The structural stability of graphene on the ASS material was tested for 28 days in simulated body fluid, and DPSCs were later seeded onto the sample. The DPSCs displayed high proliferation rates after 5 days after seeding, overall showing that the graphene-modified ASS could demonstrate potential in biomaterials that promoted bone tissue engineering for body and dental implants [107]. Another study investigated CVD-grown graphene films' ability to induce odontogenic and osteogenic differentiation of DPSCs [108]. The researchers found that graphene downregulated odontoblastic genes such as MSX-1, PAX, and DMP but upregulated osteogenic genes such as RUNX2, COL, and OCN. From these results, the authors concluded that CVD-grown graphene films may be more suited for osteogenic applications for bone tissue regeneration as opposed to endodontic and pulp regenerative applications [108]. An *in vivo* study was later performed by the same group [109] to assess if CVD-synthesized graphene scaffolds could induce osteogenesis *in vivo* and further investigate the integrin/FAK mechano-transduction pathway that potentially causes osteogenic differentiation (Figure 11). A mesenchymal stem cell-impregnated graphene scaffold was implanted into mice, and the authors discovered that the scaffold induced upregulation of RUNX2 and OPN bone related markers without the presence of osteogenic inducers. Therefore, the authors concluded that graphene scaffolds could assist in promoting osteogenic differentiation *in vivo* as well.

**Figure 12:**
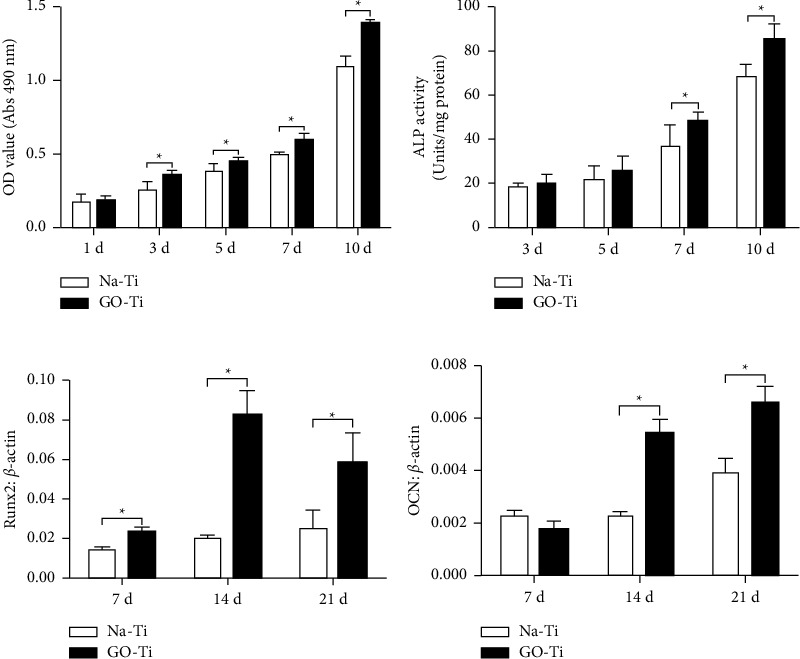
(a) MTT cell viability assay. (b) ALP activity. (c) Runx2 osteogenic gene expression in hPDLSCs seeded onto Go-Ti and Na-Ti substrates. (d) OCN osteogenic gene expression of hPDLSCs seeded onto Go-Ti and Na-Ti substrates (reprinted with permission from Zhou et al. [75] following the Creative Commons Attribution (CC BY) license (https://creativecommons.org/licenses/by/4.0/) (accessed on 4 May 2022)).

Graphene has been used in various materials to promote and support the differentiation of human periodontal ligament stem cells (hPDLSCs) as well. Graphene oxide combined with silk fibroin-based scaffolding material was found to promote hPDLSC differentiation into osteoblast-like cells [54]. GO coatings on titanate substrate materials have also been shown to display significantly higher hPDLSC proliferation rate, ALP activity, and upregulation to enhance osteogenic differentiation of hPDLSCs (Figure 12) [75]. Such studies further exhibit the potential GO-incorporated substrates can have to promote osteogenic properties on dental implants and regenerative dentistry.

Polymer substrates and scaffolds are commonly used to promote bone tissue regeneration, and graphene has been incorporated into polymer scaffolds to assist in cell proliferation, differentiation, and osteogenesis. However, most polymer substrates, on their own, possess poor mechanical properties and are hydrophobic, which decreases cell attachment and proliferation. Therefore, graphene is often added into polymer scaffolds to increase hydrophilicity and mechanical, thermal, and electrical properties [116]. A study by Qin et al. [42] modified the surface of a carbon fiber-polyether ether ketone (CF/PEEK) composite with graphene oxide to improve the bioactivity of the material. From *in vitro* tests using bone mesenchymal stem cells (BMSCs), they found that the cells had good attachment and proliferation on the GO-modified groups compared to control groups. In addition, *in vivo* studies using rat models of the material found no toxic systemic effects, and the GO-modified scaffold allowed for higher calcium deposition of the BMSCs compared to control groups [42]. Another study found that incorporating GO on PEEK-based materials allowed for better antibacterial effects against *Porphyromonas gingivalis* bacteria and promoted cell proliferation in embryonic osteoblast MC3T3-E1 cells. They also found that the addition of GO enhanced osteogenic differentiation, *in vitro,* compared to unmodified composite materials [117].

Porous polymer scaffolds are used in bone tissue engineering for orthopedic and regenerative tissue engineering in dentistry. Huang et al. [116] fabricated a 3D porous polycaprolactone/graphene (PCL/graphene) scaffold and found that osteoblast-like MG-63 cells had good viability and proliferation on the graphene added scaffolds compared to control groups. Another study included graphene into a 3D calcium silicate (CS) and PCL scaffold and tested its bone regenerative ability *in vitro* and *in vivo*. From *in vitro* tests, they found that the graphene CS/PCL scaffold displayed almost double ALP activity compared to other scaffolds, which indicates early osteoblast differentiation. From *in vivo* tests, they found that the graphene CS/PCL scaffolds promoted proliferation and osteogenesis of Wharton's jelly MSCs [118]. A hybrid porous material made of graphene and laponite was explored for its potential to induce proliferation and differentiation of MSCs. The scaffold was found to be biocompatible and allowed for homogeneous cell attachment to allow for longer term cell proliferation. Osteogenic differentiation was upregulated by inducing BMP-9 osteogenic factors [119]. The pore size and porosity of porous 3D scaffold must be considered when assisting in cell attachment and proliferation and is a main feature that affects cell attachment and proliferation. If the pores are too small, cell attachment can increase, but nutrient and gas delivery is less efficient. If the pore size is too large, cell attachment is decreased, but nutrient and gas exchange functions well. Therefore, researchers found that an appropriate pore size between 100–350 *μ*m was necessary to promote sufficient bone regeneration and osteoconductivity [116, 120].

Collagen is among the main components in soft and hard tissues and is widely distributed throughout bones, tendons, skin, and teeth [121, 122]. It is commonly used in bone tissue engineering due to its excellent biocompatibility, adhesion, osteogenic induction properties, and degradability [123]. However, using collagen on its own is not appropriate in bone tissue engineering due to its limited mechanical and physicochemical properties [124]. Therefore, collagen-based scaffolds have been modified with GO and rGO to increase mechanical stiffness and bioactivity to improve hard tissue regeneration [125]. The biocompatibility of a GO-coated collagen membrane with DPSCs was performed by Radunovic and colleagues [126]. The researchers found that GO-coated membranes increased DPSC differentiation into odontoblasts and osteoblasts by stimulating the production of BMP2 genes and RUNX2 transcriptional modulators that induced osteoblastic differentiation [126]. Another study found that GO-coated collagen scaffold enhanced MSC differentiation into osteoblasts after 14 days without addition of an osteogenic medium. In addition, apatite formation on the scaffold surface increased [125]. A study comparing rGO and GO films on collagen scaffolds found that the rGO-coated scaffolds possessed better bioactivity than GO-coated ones, but both rGO and GO allowed for successful cell attachment and differentiation of MCT3T3-E1 cells [127]. From these studies, it is clear that the addition of GO and rGO onto collagen scaffolds assists in bone tissue engineering and shows good biocompatible aspects.

Calcium hydroxyapatite (HAP) is another material used in bone tissue engineering due to its excellent biocompatibility and osteoconductivity [128]. However, on its own, it has low mechanical stability, low wear resistance, and low specific surface area [129]. Therefore, graphene oxide has been previously studied as an inclusion material to create GO/HAP composite scaffolds. One study investigated the use of GO, HAP, and agarose to test cell attachment ability of MC3T3-E1 cells and found that the scaffold increased proliferation and differentiation of cells which resulted in calcified bone tissue [104]. Another study fabricated a GO/HAP/PCL composite scaffold to also increase cell proliferation of pre-osteoblastic MC3T3-E1 cells and found that it had good biocompatibility. Increased cell proliferation occurred when GO concentrations were below 350 *μ*g/ml [36]. In addition, rGO has been used to modify biphasic calcium phosphate materials, which are a mixture of hydroxyapatite and B-tricalcium phosphate (BCP) [43]. Higher concentrations of rGO above 100 *μ*g/ml seemed to significantly decrease cell viability, but concentrations below 62.5 *μ*g/ml displayed negligible differences. However, the rGO-modified BCP coating displayed significantly higher new bone formation during *in vivo* rat studies [43].

A chitosan HAP composite modified with GO was studied to increase cell adhesion, viability, differentiation, and mineralization in osteogenic properties [105]. From *in vitro* cytological examination, researchers found that the composite had good wettability and bond strength that increased cell viability and differentiation potential for bone MSCs. *In vivo* studies also showed that osseointegration enhancement of the material was observed when implanted into rats [105]. In addition, other researchers added PEG to a chitosan/HAP-based scaffold that was GO modified and found that the addition of PEG in the scaffold may assist in increasing cell proliferation compared to scaffold that did not include PEG [130]. From these above studies, using graphene with HAP composites graphene has shown to exhibit good biocompatibility and increase cell attachment and proliferation in bone tissue engineering for dental and bone regenerative applications.

Graphene-based materials have been used as coatings for titanium implants. Various layers of graphene oxide have been added to titanium implants and have been shown to increase antibacterial properties, osteogenic cell behavior, and cell proliferation [64]. GO and silver composite coatings have been added to NiTi alloy implants to assist in biocompatibility of human pulp fibroblast cells and decrease inflammatory responses of implants. The addition of GO and silver coating on a NiTi implant increased corrosion resistance and displayed upregulation in IL-6 and IL-8anti-inflammatory cytokines [131]. GO has been used to modify titanium-based material for pulp sealing applications and allowed for dentin-like mineralization. GO-modified titanium surfaces have displayed good biocompatibility aspects by upregulating hDPSC cell adhesion, proliferation, odontogenic differentiation, and antibacterial activity [132].

In general, graphene oxide and reduced graphene oxide materials have displayed relatively good biocompatibility for various bone tissue engineering applications. The addition of graphene oxide has been shown to improve cell attachment, proliferation, and differentiation of DPSCs and hPDLSC in various porous polymer scaffolds and hydroxyapatite composites. It assists in better osseointegration of implants. However, there is still debate over the optimal concentration of GO and rGO in various scaffolds and composites to achieve optimal cell viability and cell attachment. Overall, substrates with graphene-based materials included allow for proper cell adhesion, viability, and proliferation directly related to biocompatible aspects of the overall material [102].

## 5. Conclusions and Future Perspectives

Graphene-based nanoparticles are considered a topic of great interest in the field of dentistry. They are being studied for applications such as dental adhesives, fillings, and tissue engineering. Graphene-based materials have been shown to have promising results as antibacterial agents. Antibacterial and antibiofilm properties for dental implants are crucial since bacterial infections are often the causes for implant failure and integration with the bone. The addition of graphene-based materials in dental composite fillings has offered antibacterial properties to decrease bacterial adsorption onto composite material to prevent further tooth decay. Additionally, graphene nanoparticles have been found to have adequate initial biocompatibility in the mouth when it comes to bone and tissue engineering. They have been shown to possess good osteoblastic differentiation tendencies to increase osseointegration and improve bone tissue development. Increased proliferation and differentiation of stem cells is a major factor in showing graphene's biocompatibility for dental applications.

Although graphene has been shown to display relatively good biocompatible qualities in various dental applications from *in vitro* tests, there is still a lack of long-term cytotoxicity studies of graphene nanoparticles in the body, as well as a lack of understanding as to how easily they can be excreted from the body. Many factors such as particle size, shape, and concentration appear to play a large role in how toxic these nanoparticles can be, and it would seem that the cytotoxicity varies on a case-by-case basis. Standardization of biocompatibility requirements for dental applications is also needed with long-term *in vitro* and *in vivo* studies to fully understand the biocompatibility of these nanoparticles and their long-term health effects.
